# Does the microbiome play a role in the pathogenesis of colonic diverticular disease? A systematic review

**DOI:** 10.1111/jgh.16142

**Published:** 2023-02-26

**Authors:** Raquel Cameron, Kerith Duncanson, Emily C Hoedt, Guy D Eslick, Grace L Burns, Cheenie Nieva, Simon Keely, Marjorie M Walker, Nicholas J Talley

**Affiliations:** ^1^ School of Biomedical Sciences and Pharmacy, College of Health, Medicine and Wellbeing University of Newcastle Newcastle NSW Australia; ^2^ School of Medicine and Public Health, College of Health, Medicine and Wellbeing University of Newcastle Newcastle NSW Australia; ^3^ NHMRC Centre for Research Excellence in Digestive Health University of Newcastle Newcastle NSW Australia; ^4^ Australian Gastrointestinal Research Alliance (AGIRA) Newcastle Australia; ^5^ Immune Health Research Program Hunter Medical Research Institute Newcastle Australia

**Keywords:** Colonic, Diverticular disease, Diverticulitis, Diverticulosis, Microbiota

## Abstract

**Background and Aims:**

The role of the microbiota in diverticulosis and diverticular disease is underexplored. This systematic review aimed to assess all literature pertaining to the microbiota and metabolome associations in asymptomatic diverticulosis, symptomatic uncomplicated diverticular disease (SUDD), and diverticulitis pathophysiology.

**Methods:**

Seven databases were searched for relevant studies published up to September 28, 2022. Data were screened in Covidence and extracted to Excel. Critical appraisal was undertaken using the Newcastle Ottawa Scale for case/control studies.

**Results:**

Of the 413 papers screened by title and abstract, 48 full‐text papers were reviewed in detail with 12 studies meeting the inclusion criteria. Overall, alpha and beta diversity were unchanged in diverticulosis; however, significant changes in alpha diversity were evident in diverticulitis. A similar Bacteroidetes to Firmicutes ratio compared with controls was reported across studies. The genus‐level comparisons showed no relationship with diverticular disease. Butyrate‐producing microbial species were decreased in abundance, suggesting a possible contribution to the pathogenesis of diverticular disease. *Comamonas* species was significantly increased in asymptomatic diverticulosis patients who later developed diverticulitis. Metabolome analysis reported significant differences in diverticulosis and SUDD, with upregulated uracil being the most consistent outcome in both. No significant differences were reported in the mycobiome.

**Conclusion:**

Overall, there is no convincing evidence of microbial dysbiosis in colonic diverticula to suggest that the microbiota contributes to the pathogenesis of asymptomatic diverticulosis, SUDD, or diverticular disease. Future research investigating microbiota involvement in colonic diverticula should consider an investigation of mucosa‐associated microbial changes within the colonic diverticulum itself.

## Introduction

Diverticula are “sac‐like protrusions”^1^ of the mucosa, protruding through the muscularis of the colonic wall, and are commonly observed anatomical alterations.^2^ These occur predominantly as a left‐sided disease of the descending, sigmoid, and rectosigmoid colon in Westernized countries.^3^, ^4^ Incidence and prevalence are increasing worldwide as lifespan increases^5^ and diagnostic techniques improve.^6^ Colonic diverticula are estimated to occur in approximately 17.5% of the general population,^3^ with 30% of individuals aged 60 and over and 70% aged over 80 years in Westernized countries having asymptomatic diverticula or symptomatic colonic diverticular disease.^4^

In those with diverticulosis, approximately 4% develop diverticulitis.^3^ There is a spectrum of observed manifestations,^4^ ranging from symptoms with no inflammation, symptomatic uncomplicated diverticular disease (SUDD),^7^, ^8^ to inflammation of diverticula and acute or chronic complications of acute diverticulitis to complicated or chronic diverticulitis. Acute diverticulitis is episodic but can become complicated by perforation and formation of abscesses,^7^, ^9^, ^10^ leading to disease progression further to chronic or complicated diverticulitis, including perforation, fistula involvement, peritonitis, bleeding, and obstruction.^3^

It is estimated that 95% of the gut microbiota resides in the colon,^11^ making this the richest region of the gut. The mucosa‐associated microbiota (MAM) of the colon resides on the colonic epithelial surface playing an important role in health in training the immune system, epithelial growth, and development, as well as contributing to the regulation of mucus production.^12^ Defects in mucus production may impact the integrity of the protective mucus barrier^13^ and may contribute to dysbiosis of the MAM.^8^ Dysbiosis in the colon is characterized by a decrease in microbial diversity coupled with an increase in proinflammatory species^14^ and has previously been implicated as a feature of colonic diverticula based on changes in Bacteroidetes to Firmicutes ratio reported.^15^

To date, the extent of microbiota involvement in diverticula formation is still debated. The role of dysbiosis of the diverticulum in diverticulosis is believed to involve mucosal barrier breakdown and microbial dislocation through areas of the diverticulum that have become compromised by acute or chronic inflammation, which has resulted in thinning of the epithelium or perforation, possibly causing diverticulitis.^8^ If correct, this is likely a functional contribution by the microbiota that may reflect a specific species profile, but as yet not clearly described in the literature.^16^

We, therefore, hypothesized that alterations in the microbial composition of the diverticulum mucosal community, including the mycobiome^17^ and metabolome,^8^, ^17^, ^18^ may be involved in the pathophysiology of asymptomatic diverticulosis formation, and potentially the progression of diverticulosis to diverticulitis. This systematic review aimed to comprehensively evaluate existing microbiota and metabolome data in asymptomatic and symptomatic diverticular disease to test this hypothesis.

## Methods

This review was conducted and reported per Preferred Reporting Items for Systematic Reviews and Meta‐Analyses (PRISMA) guidelines, using the PRISMA checklist (Fig. S1).^19^

### Data sources and search strategy

The electronic databases SCOPUS (OVID), MEDLINE (OVID), EMBASE (OVID), CINAHL (EBSCO), Cochrane Library (Wiley Online), Web of Science (Thomas Reuters), and Google Scholar were searched for studies of the microbiota within diverticular disease, up to and including September 28, 2022. The review protocol was registered with PROSPERO (CRD42021276795 08/10/2021). The search strategy was guided by PICO^20^ terms and is shown in Table 1. Diverticular disease for this search included any study with diverticulosis and/or diverticulitis and SUDD.

**Table 1:** Example of search table for literature scoping: Web of Science. Showing PICO search terms, results, and papers identified as relevant extracted for screening. Carried out with the help of Debbie Booth from UoN Library

Search carried out in: MEDLINE via UoN OVID			
#	Searches	Results	Identified as relevant
1	divertic* (Human)	42,873	
2	colon*	973,162	
3	#1 AND #2	16,265	
4	feces OR faeces OR stool	232,980	
5	#3 AND #4	1029	
6	micro*	4,757,030	
7	#5 AND #6	162	
8	mucosa OR biopsy OR tissue	5,748,430	
9	#3 AND #6	1,177,719	
10	#9 AND #3	473	88

### Study selection

Titles were uploaded to Covidence,^21^ and duplicates were removed before blinded screening against inclusion criteria (Table 2). Two reviewers (RC, KD) independently performed title and abstract screening, and then full‐text screening, with conflicts resolved by a third reviewer (EH) and the reason for exclusion documented.

**Table 2:** Inclusion and exclusion criteria: for abstract/title and full‐text screening of papers identified through systematic search

Inclusion	Exclusion
English language	Under 18
Human studies	Animal studies
Over age 18	Non‐English (or unable to be translated)
Microbiome studies from tissue/mucosa/biopsy/resection/feces/feaces/stool	Letters, case reports, reviews, or thesis/dissertation
Studied with no healthy controls can be included as along as control parameters include disease‐free tissue	SCAD or colitis‐associated studies

### Data extraction

Relevant data were extracted into an Excel spreadsheet and consolidated accordingly for distinguishing parameters (author, year, country published), demographics for cases and controls (colorectal cancer [CRC], adenoma patients, or diverticular disease patients paired “healthy tissue”), and covariate data for cohorts (number of participants, age [range, mean, median], sex ratios; Table 3). Table 4 provides further data on DNA extraction methods, amplification and profiling techniques, sample types (stool, rectal swab, biopsy, resection tissue), and the differences in tissue sample location in relation to the diverticula. Potential confounders extracted included antibiotic use within the 14 days before surgery and recorded bowel preparation (mechanical, oral preparation, e.g., fleet enema) (Table 4).

**Table 3:** Characteristics of included studies: investigating gut microbiota profiles in patients with diverticulosis and diverticular disease

				Cases				Controls		
First author	Year of publication	Country	Disease subtype	Cases/*n*	Age/years	Females/no.	Control type^†^	Control/*n*	Age/years	Females/no.
van Rossen	2021	Netherlands	Diverticulosis	19	66	5	No diverticulae	24	56.4	13
Alexandersson	2020	Sweden	Diverticulosis	83	62	39	No diverticulae	222	59	104
				37	63	19		104	60	53
			Diverticulitis	14	62	10		64	61	44
				8	58	7		37	57.5	32
Watanabe	2019	Japan	Diverticulitis	15	70	3	No diverticulae	28	66.2	5
Bundgaard‐Nielson	2019	Denmark	Undefined DD	104	63	49	CRC and adenoma	76	70.5	35
Jones	2018	USA	Diverticulosis	226	‐	120	No diverticulae	309	‐	187
Linninge	2018	Sweden	Undefined DD	16	68	10	No diverticulae	35	62	17
Lopetuso	2017	Italy	Undefined DD	8	53	4	Healthy	16	44	8
Schieffer	2017	USA	Diverticulitis	9	60.9	4	Paired non‐diverticula tissue	‐	‐	‐
Barbara	2016	Italy	Diverticulosis and SUDD	16	69	3	Healthy	14	52	8
				8	61	5				
Tursi	2016	Italy	Diverticulosis and SUDD	13	66	13	Healthy	16	56.4	16
				15	64.5	15				
O'Grady	2022	New Zealand	Diverticulitis	65	58	27	No diverticulae	27	46	13
Daniels	2014	Netherlands	Diverticulitis	31	57.8	11	No DD (Can have diverticulosis)	25	52.6	13

^†^ Control type: Healthy: patients with no identifiable gastrointestinal disease or symptoms; Paired normal tissue: tissue from cases obtained from non‐diverticula region of colon; No diverticulae: patients with CRC or other gastrointestinal indication for surgery but with no observed colonic diverticula.

CRC, colorectal cancer; DD, diverticular disease; SUDD, symptomatic uncomplicated diverticular disease.

**Table 4:** Sample collection and corresponding site (if applicable), preoperative antibiotics, or bowel preparation. Sample extraction and profiling approaches tabulated, along with amplified regions and taxonomic targets of interest for the studies reviewed

First author	Sample type	Tissue site	Antibiotics pre‐OT	Bowel prep pre‐OT	Extraction method	Profiling approach	Variable regions amplified	Taxonomic target	Metabolome assessment method
van Rossen	Biopsy	Adjacent	No	Unknown	bioMérieux—NucliSENS® easyMag®	IS‐Pro (16S–23S rRNA)/CE	16S–23S interspace regions	Phylum specific	NA
Alexandersson	Biopsy amd stool	Unknown and NA	Yes	Yes	MoBio's PowerMag	Illumina‐MiSeq	V3–V4	ASV	NA
Watanabe	Stool and fecalith	NA and ostia of diverticulum	No	No and yes	Not stated	qPCR & T‐RLFP	V1–V3	OTU specific	NA
Bundgaard‐Nielson	Biopsy	Adjacent and non‐diseased	Unknown	Unknown	QIAGEN AllPrep® DNA/RNA FFPE kit	Illumina‐MiSeq	V4	Species and sub‐species	NA
Jones	Biopsy	Adjacent	Unknown	Yes	QIAGEN DNeasy Blood and Tissue Kit	Illumina‐MiSeq	V3	Phylum to genus	NA
Linninge	Biopsy	Adjacent	Yes	Yes	QIAGEN EZ1 DNA Tissue Kit and Bacteria Card	T‐RLFP	V2	Singe bacteria of outcome	NA
Lopetuso	Stool	NA	No	No	QIAGEN QIAamp DNA Stool Mini Kit	GGS‐Junior Platform (454, Roche)	V1‐V3	Phylum specific	NA
Schieffer	Resection tissue	Adjacent and non‐diseased	Yes	Unknown	QIAGEN DNeasy Blood and Tissue Kit	Illumina‐barcoded/16S/18S &5.8S	V4/ITS1–2	Phylum to genus	NA
Barbara	Biopsy, stool, and urine	Adjacent and non‐diseased	No	Yes	QIAGEN DNeasy Blood and Tissue Mini Kit	HTF‐Microbi.Array/H‐NMR	V3‐V4	Genus and species specific +	H‐NMR spectroscopy
Tursi	Stool	NA	No	Unknown	QIAGEN QIAamp DNA Stool Kit	qPCR	V3‐V4	Genus and species specific	H‐NMR spectroscopy
O'Grady	Rectal swab	NA	No	No	ZymoBIOMICS DNA Kit	Illumina‐MiSeq	V3‐V4	Phylum to genus	NA
Daniels	Rectal swab	NA	Yes	Yes	bioMérieux—NucliSENS® easyMag®	IS‐Pro (16S–23S rRNA)/CE	16S–23S interspace regions	Phylum specific	NA

Studies were subgrouped based on the nature of the investigation as per diverticular disease subtypes diverticulosis, SUDD, and diverticulitis (Table 5), as well as microbiota analysis outcomes (dysregulation, or correlations of microbiota with immune dysregulation), mycobiome, and metabolome.

**Table 5:** Microbiota, mycobiome, and metabolome data extracted from the 12 papers. Studies were indicated as reporting a decrease in abundance (red and down arrow), an increase (green and up arrow) or no difference compared with controls (yellow and equal sign). Papers tabulated according to disease subtype, diversity, ratios, then according to the phylogenetic tree. Disease subtype: AD (asymptomatic diverticulosis), SUDD (symptomatic uncomplicated diverticular disease), SD (symptomatic diverticulitis or diverticular disease undefined)

*Microbiota, mycobiota, and metabolome data of all patients with diverticulae compared with patients without (or matched control samples)*															
	van Rossen 2021	Jones 2018	Barbara 2017	Tursi 2016	Alexandersson 2020	Barbara 2017	Tursi 2016	O'Grady 2022	Alexandersson 2020	Watanabe 2019	Bundgaard‐Nielsen 2019	Linninge 2018	Lopetuso 2017	Schieffer 2017	Daniels 2014
Disease subtype															
AD, SUDD, or SD	AD	AD	AD	AD	AD	SUDD	SUDD	SD	SD	SD	SD	SD	SD	SD	SD
Microbiota Alpha diversity															
Shannon	=	=						↓	=		=	=			↑
Simpson												=			
Richness/chao1		=						↓	=		=	=	↓	=	
Beta diversity															
Bray–Curtis									=						
Cosine distance	=														
Hellinger distance											=				
ANOSIM														=	
Ratio calculation															
Firmicutes/Bacteroidetes	=										=		↑		=
Order															
Enterobacteriales										↑				=	
Lactobacillales										↑				=	
Phylum															
Actinobacteria											↑				
Fusobacteria								↑							
Proteobacteria	=	↑													↑
Family															
Comamonadaceae		↑													
Enterobacteriaceae			↓			↓						↑	↓		↑
Lachnospiraceae								↓							
Lactobacillaceae						↓									
Microbacteriaceae														↑	
Porphyromonadaceae													↓		
Ruminococcaceae													↑		
Genus															
*Acinetobacter*															
*Akkermansia*			↓			↓									
*Bacteroides/Prevotella*			↑	=		↑	=	↑			=				
*Prevotella* OTU 317											↓				
*Comamonas*									↑						
*Clostridium* cl. IV			↓			↓				↑					
OTU 369										↓					
OTU 749										↑					
*Clostridium* cl. IX						↓									
*Clostridium* sub. XIVa										↓					
*OTU (Clostridium* cl. XI, sub. XIVa*)*										↑					
*OTU (Clostridium* sub. XIVa, Enterobacteriales*)*										↑					
*Corynebacterium*								↑							
*Faecalibacterium*								↓							
*Fusobacterium*						↓		↑			=				
*Lactobacilalles*				=			=			↑					
*Paraprevotella*								↑							
*Ruminococcus*								↓							
*Senegalimassilia*								↑							
Species															
*Akkermansia muciniphila*				↑			↑						↓		
*Bacteroides fragilis*													↓		
*Escherichia coli*				=			=								
*Faecalibacterium praunsnitzii*													↑		
Mycobiome															
Alpha diversity														=	
Beta diversity														=	
OTU *Exophiala* from phylum Ascomycota														↑	
Metabolome															
2‐Oxoglutarate						↓									
3‐Methylglutarate			↑			↑									
Butyrate							↓								
Choline							↓								
Glucose			↑												
Hippurate						↓									
Isovalerate				↓											
Kynurenine						↑									
*N*‐Acetyl compounds				↓		↓	↑								
Quinolate						↑	↑								
Tryptophan						↓	↓								
Tryptophan catabolites of the kynurenine pathway						↑	↑								
Unassigned saccharide X‐543			↑												
Uracil			↑			↑	↑								
U1							↑								
Valerate							↓								
Valine							↓								

### Outcome measures

The primary outcome was to identify characteristics of the microbiota of patients within the colonic diverticular disease spectrum to determine if there are distinct profiles in diverticular disease. Data extracted and tabulated were assessed for similarities between sample type, DNA extraction and profiling methodology, and results for narrative synthesis.

### Critical appraisal

Quality assessments were undertaken using the Newcastle Ottawa Scale (NOS)^22^ for case/control studies. Two reviewers (RC, CN) evaluated each study using the NOS scale (Table S1). Thresholds for converting the NOS were employed as per the Agency for Healthcare Research and Quality (AHRQ) standards of good, fair, and poor. Conflicts (8%) were assessed and resolved by a third reviewer (KD).

## Results

### Search results

**Figure 1:**
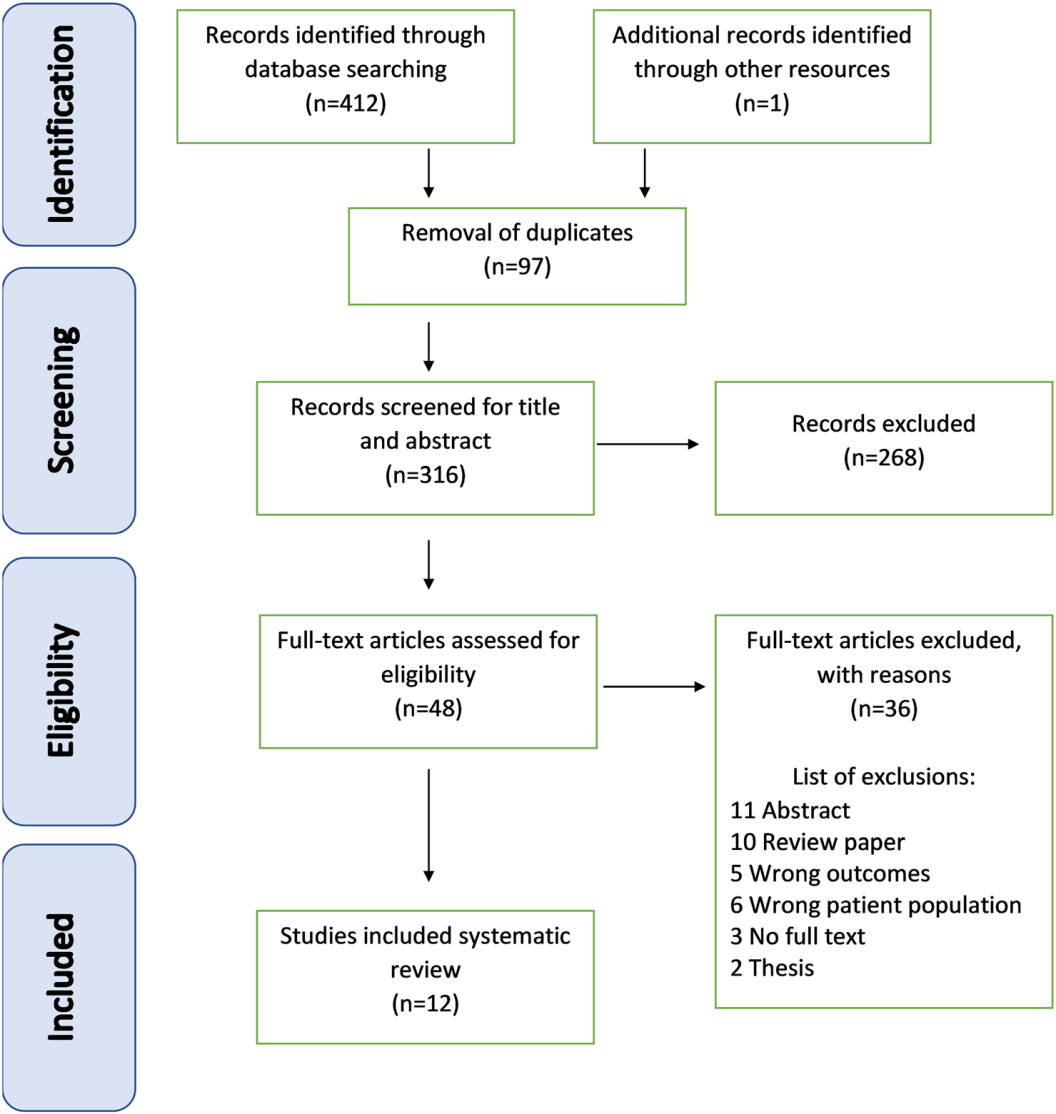
PRISMA diagram: depicting the screening methodology of papers obtained through systematically searching the literature (412), removing duplicates (97), and then screening according to inclusion and exclusion criteria (48) to obtain a final number of papers for data extraction and review analysis.^12^

Of the initial 413 articles identified (Fig. 1), 412 were identified via database search, and a further single reference was gathered via reference reading, 97 were duplicates, and 268 were not eligible for inclusion after the title and abstract screening. A further 36 papers were excluded during full‐text screening. Twelve studies remained suitable for data extraction.

### Critical appraisal outcomes

Based on the AHRQ scoring rubric (Table S2), six studies scored the highest standard of “good,”^8^, ^15^, ^23^, ^24^, ^25^, ^26^ one scored “fair,”^17^ and five scored “poor.”^16^, ^18^, ^27^, ^28^, ^29^

### Study characteristics

Sampling sites varied, with only one study analyzing the microbiota from full‐width resection samples.^17^ Six studies took colonic biopsies from non‐diverticula mucosa, five analyzed stool samples, two obtained a rectal swab, and one obtained fecalith via biopsy (Table 4). Three studies reported additional analyses of metabolome data (Table 5). Control samples were collected from asymptomatic non‐diverticula patients,^8^, ^18^ CRC, or adenoma‐diagnosed patients without diverticula,^15^, ^16^, ^17^, ^24^, ^25^, ^26^, ^27^, ^28^, ^29^ or non‐inflamed luminal tissue from the same patient (case).^17^ In one diverticulitis study,^23^ controls did not have either diverticula or diverticulosis. Eleven of the studies were specific to left‐sided disease, with only one Japanese study^28^ reporting on right‐sided diverticular disease outcomes.

### Preoperative microbiota disruption

In Table 4, presurgical antibiotic use was recorded for four^17^, ^23^, ^25^, ^26^ of the 12 studies, with the status unreported for two.^24^, ^27^ Presurgical use of fleet enemas or mechanical bowel preparation was also reported to be used in six^8^, ^23^, ^24^, ^25^, ^26^, ^28^ of the 12 studies, with the status unknown for four.^15^, ^17^, ^18^, ^27^

### Microbiota diversity is not significantly different from controls

Overall, alpha and beta diversity were not significantly different from control samples in nine studies on diverticulosis, SUDD, and diverticulitis^8^, ^15^, ^17^, ^18^, ^24^, ^25^, ^26^, ^27^ (Table 5). O'Grady *et al*.^29^ (rectal swab) and Lopetso *et al*.^16^ (stool sample) reported decreased microbial richness and alpha diversity in diverticulitis. Daniels *et al*.^23^ (rectal swab) reported the Shannon diversity increased in diverticulitis.

### Bacteroidetes/Firmicutes ratio is unchanged in diverticular disease

Included studies reported a similar Bacteroidetes to Firmicutes ratio^15^, ^23^, ^27^ compared with control samples; except for one study,^16^ which found that the ratio was significantly increased for Firmicutes and decreased for Bacteroidetes.

### Diverticulosis and SUDD study outcomes

Table 5 outlines five studies focused on asymptomatic diverticulosis,^8^, ^15^, ^18^, ^24^, ^26^ two of which also had an SUDD cohort.^8^, ^18^ The study populations varied between case numbers for diverticulosis (*n* = 13–226) and SUDD (*n* = 13–15), with a median age of 66 (range of 62–69) and 62.8 (range of 61–64.5 years), respectively. The median age of controls was 56.4 (range of 52–60 years). Controls (*n* = 659), comprising 381 female participants and 308 male participants, were patients who were defined as healthy, or who at colonoscopy were identified as having no diverticula.

Studies by Tursi *et al*.^18^ and Barbara *et al.,* ^8^ analyzing diverticulosis and SUDD patient samples, aimed to identify diverticula microbiota differences amplified at V3–V4 regions. Tursi *et al*.^18^ profiled using qPCR, and Barbara *et al*.^8^ undertook HTF‐Micro.Array that allowed targeted profiling of pre‐selected species using PCR‐based amplification methods and specific probes. Tursi *et al*.^18^ demonstrated that *Akkermansia muciniphila* species were significantly lower in stool samples from controls (−4.57 ± −1.05) than in diverticulosis cases (−3.41 ± −1.13. *P* = 0.019) and SUDD (−3.56 ± 1.27, *P* = 0.044). No other studies of diverticulosis or SUDD reported a significant difference in *A. muciniphila*. Barbara *et al*.^8^ reported a lower family‐level abundance of Enterobacteriaceae in diverticulosis and SUDD compared with control mucosal biopsy samples (20.4 ± 4.1% *vs* 34.6 ± 5.8%, *P* = 0.04) at the diverticular region, as well as a trend to higher abundance of *Bacteroides*/*Prevotella* (46.2 ± 3.3% *vs* 36.8 ± 3.2%, *P* = 0.06). In contrast, a lower abundance of genus bacteria *Akkermansia* (1.0 ± 1.0% *vs* 2.5 ± 1.1%; *P* = 0.02) was reported for SUDD compared with controls and diverticulosis. The abundance of *Clostridium* cl. IX (16.0 ± 1.8% *vs* 9.9 ± 1.5%; *P* = 0.03*)*, *Fusobacterium* (1.2 ± 0.2% *vs* 0.7 ± 0.2%; *P* = 0.05) and Lactobacillaceae (6.3 ± 1.8% *vs* 2.8 ± 0.6%; P = 0.05) were significantly decreased in SUDD compared to diverticulosis and controls.

Alexandersson *et al*.,^26^ collected sigmoid biopsy samples (*n* = 715) from diverticula adjacent tissue for correlation against fecal samples (*n* = 352). Diverticulosis samples (16S rRNA amplicon‐based sequencing Illumina‐MiSeq V3‐V4) were compared with matched control samples. No significant differences between cases and controls were reported.^26^

Jones *et al*.^24^ and Van Rossen *et al*.^15^ reported case and control cohorts for diverticulosis patient samples from colonoscopies. These studies profiled using the techniques of 16S rRNA amplicon‐based sequencing (Illumina‐MiSeq V3) for phylum to genus taxonomic specificity and IS‐pro PCR profiling to differentiate bacterial species by the length of the 16S–23S rRNA interspace region, respectively. No significant differences were reported.

Overall, the literature does not identify a distinct microbiota signature in diverticulosis or SUDD.

### Diverticulitis study outcomes

Table 5 outlines eight studies with diverticulitis microbiota outcomes.^16^, ^17^, ^23^, ^25^, ^26^, ^27^, ^28^, ^29^ The diverticulitis cases ranged from 8 to 104 participants (total *n* = 270), with a median age of 60.9 (range of 53–70) years, composed of 125 female participants and 145 male participants. Controls (*n* = 308) were composed of paired non‐diverticula tissue, CRC participants with no diverticula at colonoscopy, or healthy defined participants at colonoscopy with no obvious gastrointestinal disease. The exception was the study by Daniels *et al*.^23^ who considered patients with diverticulosis to be included with the control cohort of non‐diverticula participants. Controls had a median age of 59.3 (range of 44–70.5) years.

Sample types for diverticulitis studies were varied. Daniels *et al*.^23^ and O'Grady *et al*.^29^ collected rectal swabs; however, profiling methods varied (IS‐Pro 16S–23S interspace regions and V3–V4 regions amplified by the Illumina MiSeq, respectively). Schieffer *et al*.^17^ (Illumina‐barcoded) was the only study to analyze full‐thickness resection tissue obtained adjacent to the diverticulum for microbiota analysis, as well as comparable tissue resection samples from a non‐diseased section of the descending colon as study control samples. The other studies sampled either stool^16^, ^28^ or took biopsies at colonoscopy^25^, ^27^ by 16S T‐RLFP or Illumina‐MiSeq. Additionally, Watanabe *et al*.^28^ took biopsies of diverticula fecalith at the ostia.

The family Enterobacteriaceae had the highest taxa‐level differences between diverticulitis and control samples, predominantly for the phylum Proteobacteria (2.6[IRQ:1.07] vs.3.2[IRQ:0.5] (*P* < 0.0002)). Although the samples were profiled using IS‐pro, fleet enemas and antibiotics (undefined length of administration and type) were administered before colonoscopy, which could have altered the fecal composition in the rectum when swabs were taken during surgery.

Lopetuso *et al*.^16^ extracted and profiled DNA from stool samples via GGS‐Junior Platform (454, Roche) platform reporting taxonomy at the phylum level (V1–V3). Cases were identified as uncomplicated diverticular disease with symptomology of mild abdominal pain with (or without) bowel changes. Enterobacteriaceae and Porphyromonadaceae were almost absent in stool samples from diverticulitis cases compared with controls, irritable bowel syndrome (IBS) or inflammatory bowel disease (IBD) samples, whereas Firmicutes and Ruminococcaceae were twice as high as these comparator cohorts.

Schieffer *et al*.^17^ was the only included study to analyze the MAM from resected tissue. They used non‐inflamed tissue sections from the same patient as a control, intending to show that the microbial community differs at the site of the diverticula. The tissue extracted for profiling was from diverticula adjacent full‐thickness regions, not from within the diverticulum itself. In addition to microbial profiles (16S rRNA amplicon sequencing Illumina‐barcoded, V4 region), this study also reported mycobiome analysis (18S and 5.8S rRNA with Illumina‐NextSeq/ITS1‐2). Microbacteriaceae were significantly increased in diverticular adjacent tissue compared with distant normal tissue samples.

Alexandersson *et al*.^26^ found no significant differences in microbiota between diverticulosis cases and controls. Their study also further assessed any of the diverticulosis cohort who later developed acute diverticulitis (2.8% of the cohort). These cases now showed a significant abundance increase in the genus *Comamonas* (*P* = 0.027), compared with those who remained asymptomatic diverticulosis.

Using 16S rRNA amplicon sequencing, Bundgaard‐Nielsen *et al*.^27^ compared diverticular disease to CRC patients without diverticula to assess microbial causality. This study reported that *Acinetobacter* constituted a significant percentage of the total microbiota, as well as the increased relative abundance of *Bacteroides*, in diverticula samples.

Linninge *et al*.^25^ reported significantly higher levels of Enterobacteriaceae (*P* = 0.043) in the colonic mucosa of diverticular disease patients compared with controls.

### Right colon outcomes

The only study to address the microbiota of the right colon was published by Watanabe *et al*.^28^ Colonic diverticula are reportedly more common on the right side (ascending colon) in Asian populations.^3^ This Japanese study hypothesized that the microbiota of the feces (fecalith), within the diverticulum may be a cause of diverticulitis. Diverticulitis patient stool samples were collected, and fecalith samples were biopsied from the ostia of the diverticulum at colonoscopy. There was no difference between the microbial communities. Fecal samples in this diverticulitis cohort had significantly lower *Clostridium* cl. IV and sub. XIVa and significantly higher Lactobacillales and Enterobacteriales (T‐RLFP profiling approach, V1‐V3).

### Mycobiome study outcomes

Schieffer *et al*.^17^ (Table 5) was the only study that analyzed the mycobiome of diverticulitis patients. This study obtained full‐thickness resection tissue, analyzing diverticula‐adjacent tissue as cases, and non‐diseased resection tissue from the same patient as the control sample. The diverticulitis cases (and controls) (*n* = 9), with a median age of 60.9 years, were profiled by Illumina‐barcoded (18S and 5.8S/fungal ITS1‐2) approach. There were no significant differences between diverticular adjacent resection tissue and control tissue for either alpha or beta diversities; however, the phylum Ascomycota was enriched in diverticulitis adjacent tissue compared with the control non‐diseased tissue.

### Metabolome study outcomes

Metabolome analysis by H‐NMR spectroscopy was reported in two studies^8^, ^18^ (Table 5). Both specifically identified metabolomes associated with diverticulosis and SUDD.^8^, ^18^ Sample types differed (urine, biopsy,^8^ and stool^8^, ^18^), and findings were incongruent between diverticulosis studies. Barbara *et al*.^8^ reported that 3‐methylglutarate, glucose, and unassigned saccharide X‐543 were more concentrated in urine samples of diverticulosis patients, whereas Tursi *et al*.^18^ reported a decreased concentration of isovalerate and *N*‐acetyl compounds in diverticulosis.

SUDD patients in both studies had increased quinolate, uracil, and tryptophan catabolites of the kynurenine pathway compared with controls. In both studies,^8^, ^18^ a decreased concentration of tryptophan was reported. *N*‐Acetyl compounds were increased in one study and decreased in the other study. Both studies also reported differences in metabolite significant to their patient samples (see Table 5).

The study by Barbara *et al*.^8^ investigated a correlation between both the fecal and mucosal microbiota; and the metabolome of urine samples by conducting a functional analysis.The study was restricted to species abundance. Both the metabolome and microbiota of patients' urine were highly consistent (Coinertia analysis scoreplots). Loading plots indicate that *Clostridium* cl. IV and *Clostridium* cl. IX from feces represented the highest direct and inverse correlation to “healthy” controls.

## Discussion

This review aimed to identify differences in the microbial profile of diverticular disease compared with controls. No two studies able to be included followed the same sampling, extraction, profiling, and assessment methodology. Although there were some commonalities, the outcomes were not consistent enough between the studies to draw any solid conclusions as to whether a specific microbiota signal exists in the colon of patients with colonic diverticula.

Of particular interest, the studies included in this review suggest a decreased abundance of butyrate‐producing species may contribute to the pathogenesis of diverticular disease. Butyrate, a short‐chain fatty acid (SFCA),^30^ has been shown to have many beneficial effects in the host as an anti‐inflammatory effector on epithelial cells, a primary energy source for colonocytes, alongside a role in regulating epithelial homeostasis.^31^ Barbara *et al*.^8^ showed that the fecal microbiota was depleted of *Clostridium* cl. IV, a butyrate producer that is reported to be of increased abundance over the age of 65.^32^ This is corroborated by Tursi *et al*.^18^ who indicated that fecal butyrate was significantly reduced in SUDD, although they did not report the same reduction in *Clostridium* cl. IV or any other butyrate producers. The reduced butyrate levels in urine reported by Tursi *et al*. corroborate these results.^33^ In diverticulitis, Watanabe *et al*.^28^ found that *Clostridium* sub. XIVa was decreased in diverticulitis patients. Interestingly, this was also previously reported by Thibault *et al*.^34^ in a study on IBD patients, correlating the finding with the downregulation of butyrate transportation by MCT1 in IBD colonic mucosa, results in butyrate oxidation deficiency in intestinal inflammation.^34^ Although this could be relevant, the study undertaken by Watanabe *et al*.^28^ included mainly right‐sided diverticula, where the concentration of butyrate has been reported to be lower at the distal end of the colon (sigmoid and rectum).^35^ *Clostridium* cl. IV and *Clostridium* sub. XIVa both have functions other than butyrate production and have been reported as being associated with other pathophysiological factors such as decreased dietary fibre^31^ and slower stool transit time.^36^ Also, there is no overall corroboration that butyrate production is altered by fluctuations in the microbial community. The phyla Firmicutes encompasses several anti‐inflammatory and butyrate‐producing species, such as the commensal bacterium *Faecalibacterium prausnitzii,* ^30^ reported by Lopetso *et al*.^16^ as significantly decreased in diverticular disease compared to controls. O'Grady *et al*.^29^ found that the *Fecalibacterium* species was significantly increased in complicated compare with uncomplicated diverticulitis. As SCFAs are key metabolites and immune mediators, an increased abundance of the commensal bacterium *F. prausnitzii* ^30^ indicates an increased production of butyrate, which opposes other findings that butyrate production and *F. prausnitzii* are decreased compared with controls in diverticulosis and uncomplicated diverticulitis. These uncertainties need to be considered in future studies associating butyrate production and the disease progression of the colonic diverticulum.

One of the most interesting study outcomes came from the study by Alexandersson *et al*.^26^ where biopsy samples from diverticulosis cases were compared with controls, with no significant differences found. Cases were then followed prospectively to reassess those who went on to develop acute diverticulitis. Cases with which the disease progressed showed an increased significance in the abundance of *Comamonas*. Normally considered non‐pathogenic, this species has more recently been associated with intrabdominal infection due to colonic perforation. In a case study by Opota *et al*.,^37^ a patient with diverticulosis was diagnosed with mixed bacteremia with *Comamonas kerstersii* and *Bacteroides fragilis* . The significance is that previous profiling approaches (pre‐MALDI‐TOF era) had failed to accurately distinguish *Comamonas* species from *Pseudomonas* species.^37^ This suggests that the use of up‐to‐date profiling technology may provide an answer as to whether the species *Comamonas* rather than *Pseudomonas* are associated with the progression of diverticular disease. Note too, the data by Alexandersson *et al*. have only been published to date as an abstract,^26^ although the authors provided the full study data for our review.

There were notable limitations in the available 12 studies. The danger in extrapolating data on microbial communities away from the diverticulum itself to within the mucosa of the diverticulum is fraught when the sample type is biopsies from non‐diverticula regions or stool and rectal swabs that may not be representative of the microbiota in the diverticulum. One study biopsied the fecalith which was novel, but a sampling from the top of the fecalith may still not be representative of microbiota throughout the sac of the diverticulum. Obtaining biopsies from the diverticulum itself to assess bacterial identity and diversity of the colonic diverticula is not usually a feasible or safe option for sample collection.^17^ This can be overcome by using resection tissue that is full length, and the entire diverticulum can be assessed for microbial load or microbiota within the context of the entire diverticulum, but this also comes with limitations of needing a surgical indication for management, limiting control samples. Stool and rectal swabbing are less invasive and inexpensive proxy collection methods for obtaining samples for colonic microbial analysis.^38^ However, antibiotics and presurgical bowel preparations may hinder the understanding of the true microbial community within the sample collected. An alternative is a brush biopsy for obtaining a sample in situ during a colonoscopy.^38^ However, current methods for this need to be optimized to prevent contamination of the sample as it is removed from the colon.^15^ Regardless, a sample from within the colonic diverticulum, profiled using metagenomic shotgun sequencing to obtain species‐level microbial resolution (bacterial, archaeal, and fungal) as well as the functional capacity of the community would begin to accurately answer the question as to whether the microbiota within the diverticulum varies from the rest of the colonic MAM. However, obtaining MAM data requires adequate microbial DNA in a sample that is predominately host DNA. A further limitation, six of the study cohorts had sample sizes of 15 or less,^8^, ^16^, ^17^, ^28^ and three less than 20.^8^, ^15^, ^25^ Small sample sizes may not be a representation of the true findings and may result in a type II error.

We conclude there is no clear evidence that diverticular disease can be attributed to changes in microbial relative abundance at this time. There are a few identified differences, but at this stage, these studies are heterogenous, making it difficult to draw any agreement. This outcome is dependent solely on the methodological approaches undertaken within these studies reviewed, and future research with advances in metabolomics and metagenomic shotgun sequencing techniques of the tissue in the diverticulum bulb, neck, and ostia may lead to different conclusions.

## Supporting information


**Table S1.** A table showing the assessment criteria utilized in this review of the literature, undertaken utilizing the Newcastle Ottawa critical assessment scale for case/control studies. Studies are given a maximum of one star for each numbered item within the Selection and Exposure categories. A maximum of two stars can be given for Comparability.
**Table S2.** This table shows the final standards for the papers used in this review using the accepted thresholds for converting the Newcastle‐Ottawa scales to AHRQ standards (good, fair, and poor): Good quality: 3 or 4 stars in selection domain AND 1 or 2 stars in comparability domain AND 2 or 3 stars in outcome/exposure domain, Fair quality: 2 stars in selection domain AND 1 or 2 stars in comparability domain AND 2 or 3 stars in outcome/exposure domain, Poor quality: 0 or 1 star in selection domain OR 0 stars in comparability domain OR 0 or 1 stars in outcome/exposure domain.
**Figure S1.** PRISMA checklist.
